# Reduced serum Klotho in chronic schizophrenia: diagnostic value and association with cognitive impairment

**DOI:** 10.3389/fpsyt.2026.1918894

**Published:** 2026-08-11

**Authors:** Dongliang Liu, Huayu Ma, Man Yang, Haidong Yang, Yanling Song, Selam Wondwossen Desta, Yaozhi Liu, Xiangdong Du

**Affiliations:** 1 Xuzhou Medical University, Xuzhou, China; 2 Department of Psychiatry, Lianyungang Psychiatric Hospital, The Fourth People’s Hospital of Lianyungang, The Affiliated Hospital of KangDa College of Nanjing Medical University, Lianyungang, China; 3 Lianyungang Rehabilitation Hospital, Lianyungang, China; 4 School of Psychiatry, North Sichuan Medical College, Nanchong, China; 5 Suzhou Guangji Hospital, The Affiliated Guangji Hospital of Soochow University, Suzhou, China

**Keywords:** cognitive function, cognitive impairment, immediate memory, Klotho protein, schizophrenia

## Abstract

**Objective:**

To investigate the correlation between serum Klotho levels and cognitive function in individuals with chronic Schizophrenia (SZ).

**Methods:**

A total of 108 patients diagnosed with chronic SZ and 83 age-matched healthy controls were recruited from four psychiatric specialist hospitals in Lianyungang from January to December 2024. Serum Klotho levels were measured using an enzyme-linked immunosorbent assay. Psychopathology and cognitive function were assessed using the Positive and Negative Syndrome Scale and the Repeatable Battery for the Assessment of Neuropsychological Status (RBANS), respectively. Kendall’s tau-b correlation analyses were conducted to assess the associations between serum Klotho levels and cognitive function.

**Results:**

Compared with healthy controls, patients with SZ had significantly lower serum Klotho levels (233.61 ± 50.35 pg/mL *vs* 347.11 ± 62.64 pg/mL, *P* < 0.001). They also showed significantly lower total scores in the overall cognitive performance and across the RBANS sub-domains (all *P* < 0.01). ROC curve analysis revealed that serum Klotho levels discriminated patients with schizophrenia from healthy controls with an AUC of 0.914 (95% CI: 0.876–0.951), with an optimal cutoff of 268.25 pg/mL (sensitivity 76.9%, specificity 89.2%). After Bonferroni correction, serum Klotho levels were positively correlated with immediate memory in patients (corrected *P* = 0.010). No significant associations between serum Klotho levels and cognitive measures were found in healthy controls.

**Conclusion:**

Patients with chronic SZ have lower serum Klotho levels and show cognitive impairment, and Klotho levels are specifically associated with immediate memory in this population.

## 1 Introduction

Schizophrenia (SZ) is a chronic and severe mental disorder that primarily manifests during adolescence or early adulthood, with a lifetime prevalence of approximately 1% (1). It is characterized by a spectrum of symptoms, including positive symptoms such as hallucinations and delusions, and negative symptoms such as avolition and diminished emotional expression. It also involves significant cognitive impairment, including deficits in attention, memory, and executive function. Research indicates that cognitive impairment affects up to 80% of individuals diagnosed with SZ (2). These deficits often emerge early in the disease trajectory and persist throughout its course. Although antipsychotic drugs effectively stabilize psychotic symptoms, patients frequently experience residual cognitive dysfunction, which is associated with poor functional outcomes, considerably affecting social functioning and quality of life (3, 4).

Although conventional antipsychotic drugs can effectively alleviate positive and negative symptoms, their impact on cognitive deficits remains limited (5). The neuropathological mechanisms contributing to cognitive impairment in SZ are complex and involve multiple factors, including genetic predispositions, developmental and environmental interactions, aberrant neuronal connectivity (6), and neurotransmitter dysregulation (7). Furthermore, peripheral inflammatory cytokines (8), changes in neurotrophic factors (9), and increased oxidative stress (10) have been implicated in this process. Therefore, identifying biomarkers associated with cognitive impairment and exploring novel therapeutic targets are paramount priorities in SZ research.

In recent years, the Klotho protein has garnered considerable attention for its role in aging and neurodegenerative disorders. The Klotho gene encodes a transmembrane protein that is predominantly expressed in the choroid plexus of the brain and the kidneys, with a soluble circulating isoform also present (11). Studies have shown that Klotho exerts potent anti-aging effects, enhances N-methyl-D-aspartate (NMDA) receptor-mediated synaptic plasticity, modulates oxidative stress, and suppresses inflammatory responses (12, 13). The expression of Klotho is closely associated with the preservation of cognitive function (14). Animal studies have demonstrated that Klotho-deficient mice exhibit marked memory deficits compared with control groups (15). Moreover, upregulation of cerebral Klotho expression has been shown to reverse age-related cognitive decline and oxidative damage (16). Furthermore, a single injection of Klotho significantly improves spatial and working memory in aged non-human primates, with effects persisting for up to 2 weeks (17). In the context of neurological disorders, reduced Klotho levels correlate with cognitive deterioration in Alzheimer’s disease (AD) and Parkinson’s disease, while its overexpression has been found to enhance cognition through mechanisms such as improved synaptic plasticity and attenuated β-amyloid toxicity (18–20).

Several hypotheses have been proposed to explain the pathological mechanisms underlying cognitive impairment in AD, among which two core pathways have been widely recognized as key drivers of disease progression. The first involves glutamatergic excitotoxicity and NMDA receptor dysfunction, where excessive glutamate can trigger neuronal calcium influx and apoptosis, and aberrant NMDA receptor function is also strongly implicated in AD-related cognitive decline (21, 22). The second is the neurotoxic cascade induced by β-amyloid protein, which initiates a robust neuroinflammatory response and drives widespread synaptic loss, ultimately contributing to progressive cognitive impairment (23, 24). Notably, these core pathological mechanisms in AD, particularly NMDA receptor dysfunction and neuroinflammatory damage, are closely aligned with the known protective targets of the Klotho protein, which exerts neuroprotective and cognition-enhancing effects. Interestingly, similar pathological mechanisms have also been implicated in cognitive impairment in SZ. Current research indicates that cognitive deficits in SZ are closely associated with NMDA receptor hypofunction (25), which affects learning, memory, and executive processes, thereby forming the basis of the glutamate hypothesis of the disorder (26, 27). Furthermore, reduced hippocampal neurogenesis (28), elevated oxidative stress (29), and a persistent pro-inflammatory state (30) have all been linked to cognitive impairment in SZ.

Importantly, these pathological processes represent central targets through which Klotho exerts its neuroprotective effects. Based on this overlap in pathological mechanisms and Klotho’s protective functions, we hypothesize that aberrant expression of Klotho in SZ may diminish neuroprotection and reduce the capacity to counteract oxidative and inflammatory damage, thereby contributing to cognitive dysfunction.

However, the role of Klotho in SZ remains unclear. A study by Xiong et al (31) demonstrated elevated levels of Klotho protein in the plasma of patients with SZ and identified a positive association between plasma Klotho concentrations and cognitive function, suggesting that Klotho may play a compensatory role in cognitive preservation. In another study, Turkmen et al (32) observed an association between Klotho and cognitive performance in patients with acute SZ. Despite these observations, research focusing specifically on the role of Klotho in SZ remains relatively limited, and the reported findings have been inconsistent.

This study focuses on patients with chronic SZ, a population in which cognitive deficits are often more severe, persistent, and strongly associated with long-term functional impairment. We sought to characterize serum Klotho levels in these patients and systematically evaluate their relationship with cognitive function across multiple domains, aiming to identify any significant associations after controlling for relevant confounders. In particular, we sought to assess cognitive function in patients with chronic SZ, quantify their serum Klotho protein levels, and analyze the correlations among Klotho concentrations, clinical symptoms, and cognitive performance. Through this approach, we aimed to provide clearer insights into the role of Klotho in chronic SZ and evaluate its potential utility as a biomarker related to cognitive status.

## 2 Methods

### 2.1 Participants

From January to December 2024, a total of 108 patients with chronic SZ who were hospitalized for a prolonged duration were recruited from four psychiatric specialty hospitals in Lianyungang, China (1): The Fourth People’s Hospital of Lianyungang, (2) Lianyungang Rehabilitation Hospital, (3) Ganyu District Rehabilitation Hospital of Lianyungang, and (4) Guanyun County Psychiatric Hospital of Lianyungang. Inclusion criteria for the patient group were as follows: (1) Fulfilment of the Diagnostic and Statistical Manual of Mental Disorders, Fifth Edition criteria for SZ; (2) Disease duration > 2 years, with stable antipsychotic treatment for ≥ 12 months; (3) Han Chinese ethnicity; and (4) Age between 18 and 60 years. Exclusion criteria were as follows: (1) Severe physical disorders (*e.g.*, organic brain diseases, endocrine diseases, abnormal blood test results, or cardiac, hepatic, or renal dysfunction; (2) A history of intellectual disability or alcohol or substance dependence; (3) Use of immunomodulators, neurotrophic agents, or antioxidants; and (4) Receipt of electroconvulsive therapy or transcranial magnetic stimulation within the past 6 months.

The healthy controls (HCs) consisted of 83 age-, sex-, and ethnicity-matched individuals recruited from the same region during the same period. Inclusion criteria for controls were: (1) No personal or family history of psychiatric disorders; and (2) No history of substance abuse, traumatic brain injury, neurological disorders, chronic medical conditions, or current medication use.

### 2.2 Data collection

#### 2.2.1 Demographic and clinical data

Psychiatrists collected demographic data, including sex, age, height, weight, years of education, smoking status, and medication history. Antipsychotic dosages were converted to chlorpromazine (CPZ)-equivalent doses (33). The severity of patients’ symptoms was assessed using the Positive and Negative Syndrome Scale (PANSS).

#### 2.2.2 Serum Klotho levels

**Blood collection, processing, and storage:** Fasting peripheral blood samples (5 mL × 2) were collected between 7:00 AM and 9:00 AM on the day of cognitive assessment. The samples were centrifuged at 2000 × *g* for 10 min, and the serum was aliquoted and stored at −80 °C until batch analysis.

**Assay method:** Serum Klotho protein concentrations (pg/mL) were measured using an enzyme-linked immunosorbent assay by Nanjing Byabscience Biotechnology Co., Ltd. (Nanjing, China). To minimize the risk of batch-related confounding, samples from patients with schizophrenia and healthy controls were randomly interleaved across microplate wells rather than being assayed as separate, group-segregated batches, so that each plate contained a balanced mixture of case and control samples. All samples were processed on the same day using reagents from a single lot/batch, ensuring identical assay conditions across the entire cohort. Laboratory personnel performing the ELISA and reading the plates were blinded to each sample’s diagnostic group throughout sample handling and measurement, and group allocation was only linked to the results after all measurements were completed.

#### 2.2.3 Cognitive assessment

Cognitive function was evaluated using the Repeatable Battery for the Assessment of Neuropsychological Status (RBANS) by trained psychiatrists. Assessments were conducted in the morning and required approximately 20–30 min for each participant. The RBANS comprises five subscales, namely Immediate Memory, Visuospatial/Constructional, Language, Attention, and Delayed Memory, and a total score. Raw scores were converted into index scores for analysis, with higher scores indicating better cognitive performance.

#### 2.2.4 Statistical analysis

Data were analyzed using SPSS version 28.0 (IBM Corp., Armonk, NY, United States). The normality of continuous data was assessed using the Shapiro–Wilk test. Categorical variables were analyzed using the *χ²* test. Continuous variables were expressed as mean ± standard deviation (*x̄* ± *s*) if normally distributed, and median and interquartile range if non-normally distributed. Between-group comparisons were conducted based on data distribution: The independent-samples *t*-test was used for parametric data, and the Mann–Whitney *U* test was used for non-parametric data. Between-group differences in RBANS total and subscale scores were analyzed using multivariate analysis of variance (MANOVA). Bonferroni correction was applied to the five subscale scores (initial *P* values multiplied by 5).

In the SZ group, correlations between serum Klotho levels and cognitive function were examined using Kendall’s tau-b correlation analyses across the five RBANS subscale scores and the total score, given that the subscale scores were treated as ordinal variables with a limited number of discrete values. Bonferroni correction was applied to control the risk of Type I error across the five subscale comparisons. Multivariate linear regression analysis was conducted to assess the independent association between antipsychotic regimen and serum Klotho levels, after adjusting for age, sex, disease duration, smoking status, and body mass index (BMI). Intergroup differences in RBANS cognitive scores were examined using the Mann–Whitney *U* test. Receiver operating characteristic (ROC) curve analysis was performed to evaluate the diagnostic performance of serum Klotho in discriminating patients with schizophrenia from healthy controls. The optimal cutoff value was determined by the maximum Youden index. The area under the curve (AUC) with 95% confidence interval was reported.

In addition, to assess the influence of demographic factors, serum Klotho levels were compared between smokers and non-smokers, as well as between male and female patients, using independent-samples *t*-tests. To investigate the specific effects of antipsychotic medications, numerous supplemental analyses were conducted. The association between antipsychotic dosage (in CPZ-equivalent dose) and Klotho levels or RBANS scores was assessed using Pearson correlation. Demographic and clinical characteristics between monotherapy and polytherapy groups were compared using appropriate tests based on data distribution. Differences in serum Klotho levels across the four hospitals were compared using the Kruskal–Wallis one-way analysis of variance.

In the HC group, the same Kendall’s tau-b correlation analyses were performed to examine the associations between serum Klotho levels and the five RBANS subscale scores and the total score. No Bonferroni correction was applied, as the primary hypothesis focused on the SZ group. Statistical significance was set at *P* < 0.05 (with all tests two-tailed).

## 3 Results

### 3.1 Baseline characteristics and intergroup comparisons

The clinical, demographic, cognitive, and biomarker profiles of the participants are summarized in **Table 1**. The groups were comparable across all demographic variables (all *P* > 0.05). Multivariate analysis of covariance, adjusted for sex, age, years of education, BMI, and smoking status, revealed that patients with SZ had significantly lower scores across all RBANS cognitive domains and the total score (all *P* < 0.001).

**Table 1:** Demographic, clinical, cognitive characteristics, and serum Klotho levels in schizophrenia patients and healthy controls.

	SZ Patients (*n* = 108)	HCs (*n* = 83)	Statistics	*P*
Demographic and clinical factors				
Gender (Male/Female)	34/74	28/55	*χ²* = 0.109	0.742
Age (years)	42.73 ± 9.68	41.83 ± 9.13	*t* = 0.653	0.515
Education (years)	8.81 ± 3.66	9.43 ± 2.70	*t* = -1.313	0.191
BMI (kg/m²)	24.81 ± 4.08	24.26 ± 3.56	*t* = 0.970	0.333
Smoking status (Yes/No)	17/91	15/68	*χ²* = 0.183	0.669
Disease duration (months)	204.74 ± 112.24	N/A	N/A	N/A
Age at onset (years)	26.32 ± 8.96	N/A	N/A	N/A
CPZ equivalents (mg/day)	530.48 ± 313.52	N/A	N/A	N/A
Positive symptoms score	14.29 ± 6.65	N/A	N/A	N/A
Negative symptoms score	17.63 ± 6.62	N/A	N/A	N/A
General psychopathology score	32.02 ± 8.08	N/A	N/A	N/A
PANSS total score	63.94 ± 17.16	N/A	N/A	N/A
RBANS domain				
Immediate Memory Visuospatial/Constructional Language Attention Delayed Memory	51.94 ± 13.03 76.52 ± 18.26 58.81 ± 15.48 77.57 ± 18.11 59.85 ± 18.49	79.84 ± 18.17 89.10 ± 14.63 94.60 ± 13.57 106.06 ± 16.46 83.37 ± 16.84	*F* = 32.55 *F* = 7.05 *F* = 49.37 *F* = 25.73 *F* = 20.03	**<0.001 **<0.001 **<0.001 **<0.001 **<0.001
RBANS total scores	58.40 ± 12.53	87.45 ± 13.90	*F* = 46.86	**<0.001
Biomarker				
Klotho (pg/mL)	233.61 ± 50.35	347.11 ± 62.64	*t* = -13.879	**<0.001

Data are presented as mean ± standard deviation (SD) or number of participants (*n*). Group comparisons for demographic variables were performed using independent-samples *t*-tests or χ² tests, as appropriate. Cognitive outcomes [Repeatable Battery for the Assessment of Neuropsychological Status (RBANS) domains and total score] were analyzed using multivariate analysis of covariance, and serum Klotho levels were analyzed using analysis of covariance, with adjustment for sex, age, years of education, body mass index (BMI), and smoking status. Reported *F* and *P* values correspond to adjusted models. All covariates had variance inflation factors < 2.0, indicating no significant multicollinearity. **P < 0.001. CPZ: Chlorpromazine; HCs: Healthy controls; N/A: Not applicable; PANSS: Positive and Negative Syndrome Scale; SZ: Schizophrenia.

### 3.2 Significantly lower serum Klotho levels in SZ patients

**Figure 1:**
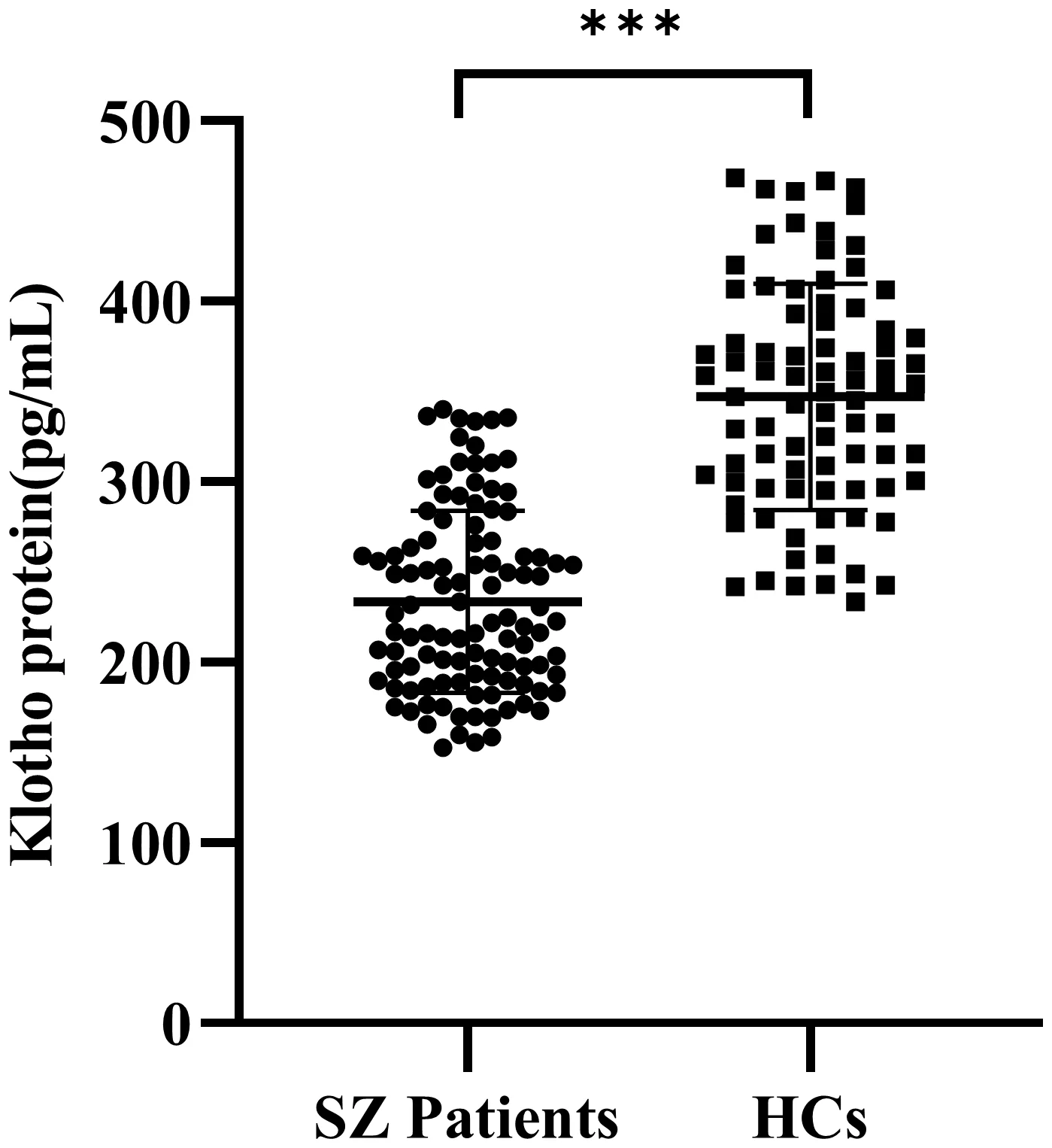
Serum Klotho levels in patients with schizophrenia and healthy controls. Serum Klotho concentrations were significantly lower in patients with schizophrenia (SZ) compared with healthy controls (HCs). Individual data points are shown for each participant. The horizontal bars represent mean values, and the error bars indicate the standard deviation. Statistical significance was determined using an unpaired *t*-test with Welch’s correction (***P < 0.001).

With regard to biomarker levels, serum Klotho levels were also significantly lower in patients compared with controls. This reduction is visually presented in **Figure 1** and was confirmed by an unpaired *t*-test (*P* < 0.001). Furthermore, analysis of covariance, adjusting for the same covariates, yielded a significant adjusted mean difference [−114.33 pg/mL; 95% confidence interval (CI): −130.53 to −98.14; *P* < 0.001].

### 3.3 Correlations between serum Klotho levels and cognitive variables

After ruling out potential confounding effects of key clinical variables, the correlation between serum Klotho levels and cognitive function was analyzed using Kendall’s tau-b correlation, given that the RBANS subdomain scores are ordinal variables with a limited number of discrete values. Klotho levels were positively correlated with RBANS total scores (τ = 0.186, *P* = 0.005), specifically within the Immediate Memory (τ = 0.220, *P* = 0.002), Attention (τ = 0.151, *P* = 0.024), and Delayed Memory (τ = 0.153, *P* = 0.024) domains. No significant associations were found with the Visuospatial/Constructional (τ = 0.105, *P* = 0.116) or Language (τ = 0.106, *P* = 0.114) domains. However, following Bonferroni correction for multiple comparisons, only the correlation between serum Klotho levels and the Immediate Memory domain remained statistically significant (corrected *P* = 0.010; **Table 2**). Conversely, in the HC group, no significant associations were observed between serum Klotho levels and either demographic variables or cognitive measures.

**Table 2:** Kendall’s tau-b correlation between serum Klotho levels and RBANS cognitive domain scores in patients with schizophrenia (*n* = 108).

Cognitive domain	Kendall’s τ	*P*	*P* (Bonferroni-corrected)
RBANS total score	0.186	0.005	—
Immediate Memory	0.220	0.002	0.010
Visuospatial/Constructional	0.105	0.116	0.580
Language	0.106	0.114	0.570
Attention	0.151	0.024	0.120
Delayed Memory	0.153	0.024	0.120

Bonferroni correction was applied for five comparisons (Immediate Memory, Visuospatial/Constructional, Language, Attention, and Delayed Memory subdomains); the RBANS total score was not included in the correction as it represents a composite index rather than an independent subdomain comparison.

**Klotho as an independent predictor of immediate memory in multiple linear regression**.

**Figure 2:**
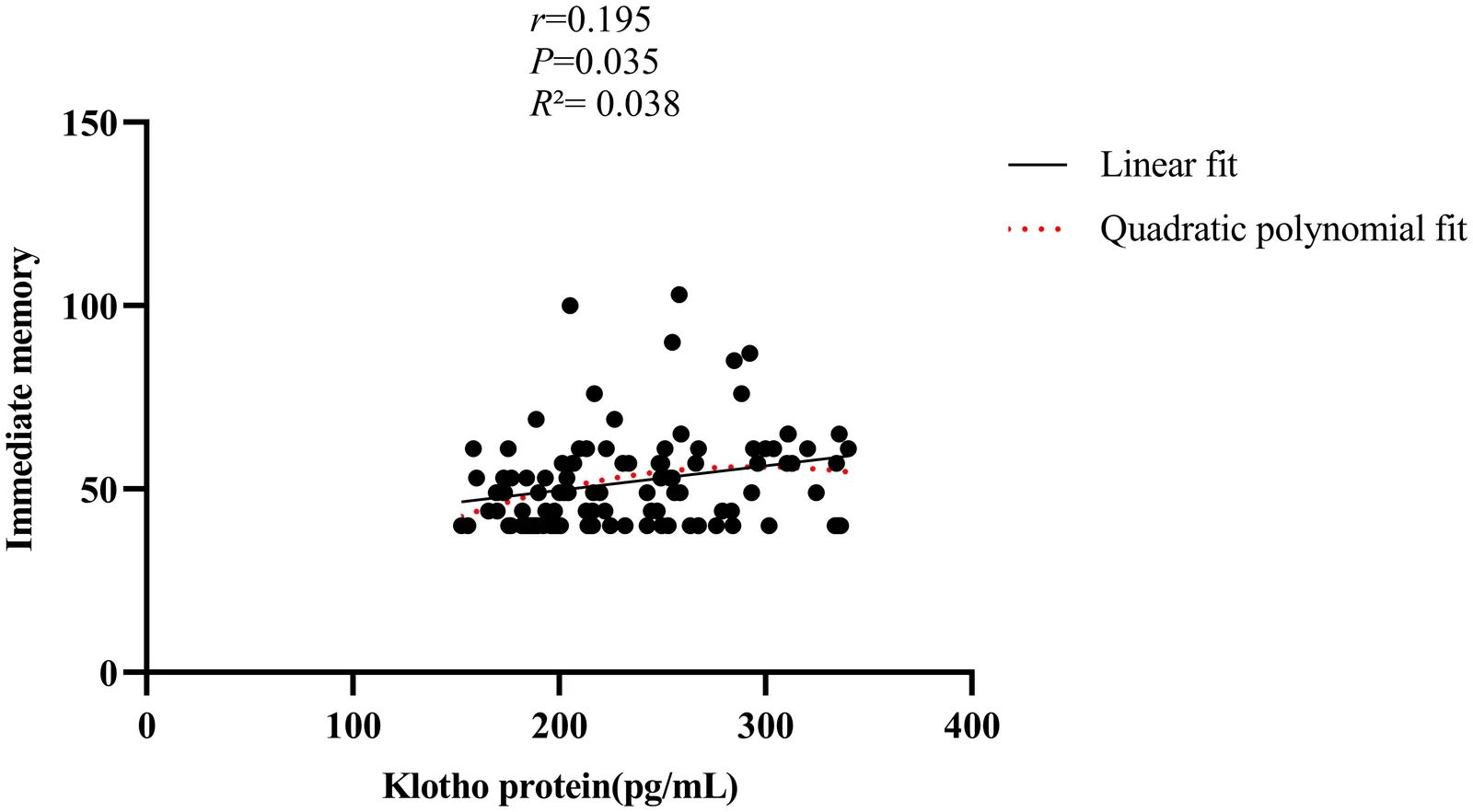
Association between serum Klotho levels and immediate memory in SZ group. The scatter plot displays individual data points for all patients (*n* = 108). The solid line represents the linear regression fit (*r* = 0.195, *R*² = 0.038), and the dashed line represents the quadratic polynomial regression fit.

Further stepwise multiple regression analysis demonstrated that the association between serum Klotho levels and immediate memory remained significant after adjusting for potential confounding factors, including sex, age, BMI, smoking status, years of education, age at onset, disease duration, and antipsychotic medication dosage. The overall full regression model, which included serum Klotho levels and all the aforementioned covariates, was statistically significant (*F* (9, 98) = 3.730, *P* < 0.001) and explained 18.7% of the variance in immediate memory scores (adjusted *R*² = 0.187). Within this model, serum Klotho levels were independently associated with immediate memory performance in patients with SZ (*β* = 0.195, *t* = 2.141, *P* = 0.035). To further investigate the potential nonlinear relationship between serum Klotho protein concentration and immediate memory score, quadratic polynomial regression analysis was conducted. Although the overall model was statistically significant (*F* (2, 105) = 4.725, *P* = 0.011), it explained only a small proportion of variance (adjusted *R²* = 0.065). Moreover, the quadratic coefficient was negative (B_2_ = −0.001), with a 95%CI including zero (−0.002 to 0.000), suggesting no meaningful nonlinear association (*t* = −1.354, *P* = 0.179). Collectively, these analyses support a persistent, significant linear association between serum Klotho levels and immediate memory performance after controlling for relevant confounders (**Figure 2**).

### 3.4 Diagnostic performance of serum klotho for distinguishing patients with schizophrenia from healthy controls

**Figure 3:**
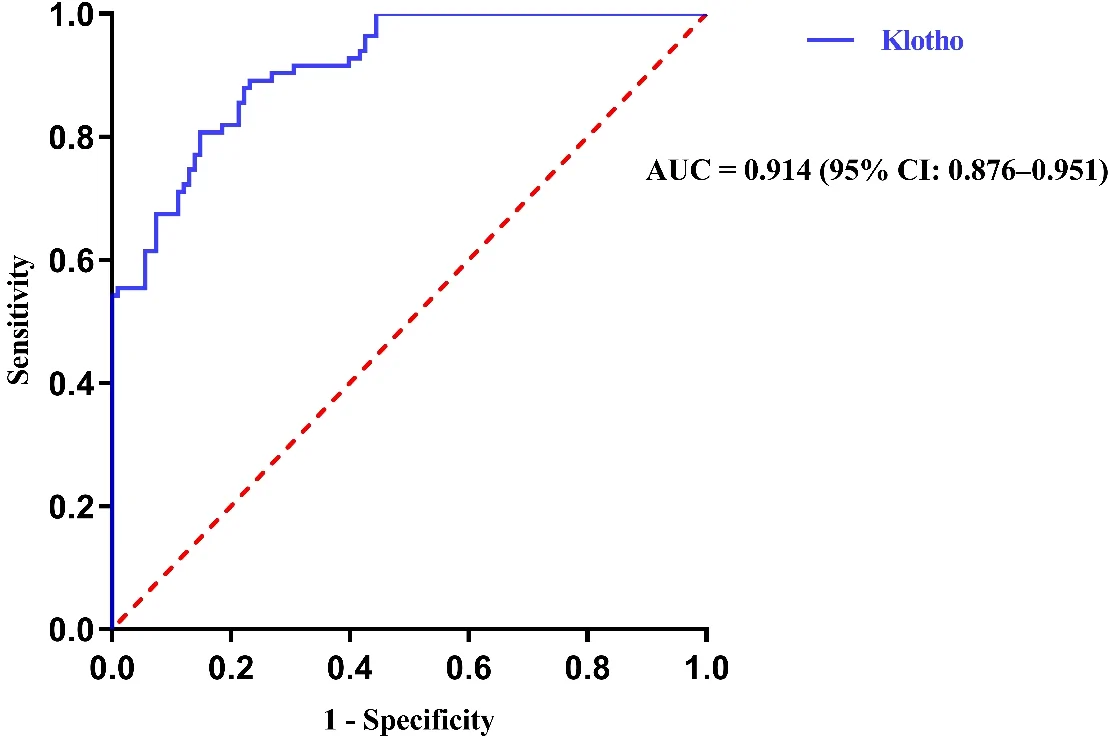
ROC curve of serum Klotho for distinguishing patients with schizophrenia from healthy controls.

ROC curve analysis demonstrated that serum Klotho levels could significantly discriminate patients with schizophrenia from healthy controls, with an AUC of 0.914 (95% CI: 0.876–0.951, *P* < 0.001), indicating excellent discriminative ability. The optimal cutoff value was determined to be 268.25 pg/mL based on the maximum Youden index, yielding a sensitivity of 76.9% and a specificity of 89.2% (**Figure 3**). Bootstrap resampling (2,000 iterations) confirmed the stability of the ROC analysis, yielding a consistent AUC (median = 0.915, 95% CI: 0.875–0.947) and optimal cutoff (median = 276.0 pg/mL, 95% CI: 258.6–312.8 pg/mL).

### 3.5 Analyses of potential confounding variables

To examine potential confounding effects, we conducted a series of supplementary analyses. Within both groups, serum Klotho levels did not differ significantly by sex or smoking status (all *P* > 0.05, **Supplementary Figure 1**). In the SZ group, Klotho levels showed no significant correlations with age, BMI, years of education, age at onset, duration of illness, CPZ-equivalent dose, or any PANSS subscale scores (all *P* > 0.05). Furthermore, no significant differences in Klotho levels or cognitive performance were observed between patients on monotherapy versus polytherapy (all *P* > 0.05; **Supplementary Table 1**). Additionally, serum Klotho levels did not differ significantly across the four participating psychiatric hospitals (*H* = 1.558, df = 3, *P* = 0.669; **Supplementary Table 2**). These findings indicate that the association between serum Klotho and immediate memory was not attributable to demographic characteristics, illness severity, antipsychotic treatment regimen, or hospital-specific factors.

## 4 Discussion

This study demonstrated that patients with SZ exhibited significantly lower RBANS total and subscale scores compared with HCs. Additionally, serum Klotho protein levels were significantly reduced in the patient group. Furthermore, serum Klotho protein levels were positively associated with immediate memory performance in the patients.

This study focused on patients with chronic SZ, systematically investigating serum Klotho protein expression and its relationship with clinical symptoms and cognitive function. Our findings revealed that the patient group exhibited significantly lower RBANS total and subscale scores compared with the HC group, indicating the presence of extensive and significant cognitive impairment. This result is consistent with numerous previous studies on cognitive impairment in SZ (34–36). More importantly, it further confirms that such impairments persist even during relatively stable remission phases of the illness. This highlights the persistent and independent nature of cognitive dysfunction as a core pathological feature of chronic SZ, rather than a transient outcome of acute symptom exacerbation. From a neurobiological perspective, cognitive impairment in chronic SZ is closely associated with abnormalities across multiple brain systems. Structural abnormalities and weakened functional connectivity in the prefrontal cortex and hippocampus, particularly the reduced signaling efficacy of dopamine D1 receptors in the dorsolateral prefrontal cortex, directly impair higher cognitive functions such as working memory and executive control (37). Furthermore, functional dysregulation in the temporal–prefrontal circuit and abnormalities in glutamatergic neurotransmission may impair neuronal synchronization and reduce the efficiency of information processing, which underlie attention and memory deficits (38).

A reciprocal relationship exists between chronic oxidative stress and chronic inflammation, which leads to impaired apoptosis and neurogenesis processes, as well as the development of neurodegenerative changes (10, 30). These mechanisms play a significant role in the etiology of SZ. It is noteworthy that these impairments at the neural circuit and molecular levels often interact with and influence each other. Therefore, SZ is a disorder that results from the interaction of multiple systems and mechanisms. This complexity may partly explain why current pharmacological and non-pharmacological interventions show limited efficacy in improving long-term functional outcomes.

Furthermore, this study observed significantly lower serum Klotho levels in patients with SZ compared with HCs, a finding that is inconsistent with some previous reports. For instance, Xiong et al (31) observed elevated plasma Klotho levels in patients with acute SZ, which correlated with cognitive function, proposing that Klotho may act as a compensatory protective factor for cognition. Although that study measured plasma rather than serum Klotho, both measures are generally considered to reflect soluble Klotho levels in peripheral circulation. We acknowledge that serum and plasma Klotho concentrations may not be directly interchangeable, potentially owing to inherent differences in sample processing procedures and specimen characteristics.

However, two critical considerations argue against this difference, explaining the discrepant findings. First, methodological studies have demonstrated that reliable immunoassays can achieve consistency between serum and EDTA plasma measurements. For example, Heijboer et al (39) reported a linear correlation (*R*² = 0.99) between serum and EDTA plasma samples when using a properly validated method, indicating that the matrix-related variability can be minimized. Second, and more importantly, any systematic difference between serum and plasma measurements would apply uniformly to both patient and control groups. Such an effect could alter the absolute concentrations but would be unlikely to reverse the direction of the relative difference between groups.

Taken together, the stark contrast between our findings and those of Xiong et al (31)likely reflects a biological distinction rather than a methodological artifact. Nevertheless, whether this discrepancy is attributable to differences in disease phase (chronic vs. acute) remains a tentative hypothesis that cannot be definitively confirmed within our cross-sectional study framework. Similarly, several studies suggest that Klotho may undergo compensatory increase under conditions of acute stress. For example, in animal models of acute myocardial infarction (40) and cardiorenal syndrome induced by renal ischemia–reperfusion (41), Klotho exerts protective effects by suppressing inflammation or enhancing FGF23 signaling. In an acute pancreatitis model, Klotho also reduced IL-6 and TNF-α levels, demonstrating anti-inflammatory potential (42).

In this context, the physiological stress or neurotoxic challenges associated with acute SZ could theoretically trigger a transient upregulation of Klotho to mitigate neuroinflammation and cognitive impairment. However, findings in clinical populations remain inconsistent. For example, Turkmen et al (32) found lower serum Klotho levels in drug-free patients during acute episodes, with a slight but statistically nonsignificant increase by day 20 of treatment. The authors suggested these results indicate a dynamic relationship between SZ and Klotho levels, although the exact reasons for the fluctuations remain unclear.

In contrast, Yazici et al (43) observed a nonsignificant increasing trend in Klotho levels among acutely ill patients. Taken together, these studies indicate that changes in Klotho levels during acute SZ are inconsistent and may be influenced by multiple factors, including medication status, illness severity, and the timing of measurement, reflecting the highly complex and dynamic nature of Klotho regulation in the acute phase.

The patients enrolled in the present study were in a clinically stable chronic phase of SZ. Our findings indicate that this group exhibits consistently lower serum Klotho levels compared with HCs. This observation is consistent with previous reports indicating that the Klotho protein may become depleted in various chronic conditions. For instance, in chronic obstructive pulmonary disease, Klotho expression was found to be reduced in the airway epithelium (44), demonstrating its downregulation at the tissue level. Complementing this finding, studies in systemic sclerosis, which is another chronic somatic disease, have demonstrated lower circulating serum Klotho levels in affected patients compared with HCs (45). A study on individuals experiencing chronic psychological stress demonstrated that those with higher stress levels exhibited lower Klotho levels (46). These findings may suggest that Klotho depletion could represent a common pathophysiological pathway underlying various chronic conditions. The underlying mechanism is likely associated with long-term pathophysiological processes, including systemic low-grade inflammation, oxidative stress, and metabolic disturbances, which have been reported to suppress Klotho expression (47, 48). Further large-scale longitudinal studies that cover the entire disease course are required to elucidate the dynamic changes of Klotho levels across different illness stages and to clarify whether its reduction represents a consequence of chronicity or a contributing factor to disease progression.

In contrast, Kazgan et al (49) reported elevated serum Klotho levels in patients with SZ. However, as that study did not specify the disease stage of the participants and included both medicated and unmedicated patients (33 *vs* 7 cases), the interpretation of its results is constrained by multiple confounding factors. It is therefore difficult to attribute the observed changes in Klotho solely to the disease itself, pharmacological intervention, or their interaction. In fact, previous studies have suggested that medications can modulate both Klotho expression and cognitive impairments associated with its deficiency. For example, a study (50) demonstrated that melatonin alleviates oxidative stress and related memory deficits caused by Klotho deficiency through interactions between the MT2 receptor and antioxidant signaling pathways involving ERK and Nrf2. Similarly, another study (51) demonstrated that low-dose fluvastatin and valsartan, either alone or in combination, significantly increased Klotho gene expression. These findings indicate that pharmacological interventions may directly influence Klotho protein levels.

However, our findings indicate that antipsychotic medications exert limited independent effects on Klotho regulation within the current study population. Specifically, no significant associations were observed between antipsychotic dosage and either serum Klotho levels or cognitive performance, nor were there significant differences between monotherapy and polytherapy regimens, with all effect sizes being small. Nevertheless, several methodological considerations should be acknowledged when interpreting these null findings. First, the exposure assessment indicators employed in this study, including chlorpromazine equivalent dosage and classification of monotherapy versus combination therapy, may not fully capture the impacts of cumulative treatment history, anticholinergic burden, treatment duration, or the potential differential effects of specific drug subclasses. Second, the comparison between typical and atypical antipsychotics was constrained by limited statistical power, as only 15 patients were receiving typical agents, most of whom were receiving combination therapy. Consequently, this particular comparison should be interpreted with caution, and the possibility of undetected between-group differences cannot be entirely excluded.

The ancillary analysis revealed no significant differences in Klotho levels across the four hospitals, suggesting that hospital site did not materially confound our primary findings. Although all four hospitals are located within the same prefecture-level city, which may reduce the impact of climatic and environmental variations, we cannot rule out the potential influence of unmeasured factors such as dietary habits, daily routines, or socioeconomic status. However, the sample size for each individual hospital was relatively small (ranging from 17 to 39), which limited the statistical power to detect subtle between-site differences, and these unmeasured factors warrant further investigation in larger, more diverse cohorts.

Regarding the association between Klotho and clinical symptoms, this study found no significant correlation between serum Klotho levels and the core clinical manifestations of SZ, including positive and negative symptoms. This result is consistent with the findings reported by Turkmen et al (32) and Xiong et al (31). To date, no published studies have clearly established a significant association between Klotho levels and symptom severity in SZ. Given the relatively limited number of investigations in this area, further research is needed to confirm whether Klotho expression is indeed related to clinical symptoms.

The ROC curve analysis in the present study demonstrated that serum Klotho levels exhibited excellent discriminative ability in distinguishing patients with schizophrenia from healthy controls, with an AUC of 0.914. These findings suggest that reduced serum Klotho may serve as a potential peripheral biomarker for schizophrenia. The sensitivity suggests that a substantial majority of patients in the current sample had Klotho levels below this threshold, consistent with the significantly lower Klotho concentrations observed in the schizophrenia group compared with healthy controls. However, despite the relatively high specificity, low Klotho levels alone are insufficient for definitive diagnosis, and Klotho should be considered as a complementary rather than standalone biomarker. Furthermore, given that the sensitivity and specificity values may partly reflect the characteristics of the current sample, external validation in larger, independent cohorts is warranted before clinical application.

A key finding of this study was the observed statistically significant correlation between reduced serum Klotho levels and impaired immediate memory performance, indicating that lower Klotho levels are associated with worse immediate memory. This suggests that low Klotho levels may be closely related to deficits in specific cognitive domains in patients with chronic SZ. Cognitive impairment in SZ is strongly associated with mechanisms such as oxidative stress (52), hypofunction of the NMDA receptor (53), and impaired synaptic plasticity (54). Klotho protein, recognized as a neuroprotective factor, is thought to maintain synaptic plasticity and cognitive function through its high expression levels, which enhance antioxidant defense mechanisms (16) and modulate NMDA receptor function—particularly by promoting the retention and functionality of GluN2B subunits at synapses in the hippocampus and prefrontal cortex (16, 55). These mechanisms are essential for the functional integrity of the hippocampus and prefrontal cortex (56), which are brain regions critically involved in supporting cognitive domains such as memory, attention, and executive function.

Within the framework of existing biological evidence, our finding of lower Klotho levels may reflect a state of reduced capacity to counteract oxidative damage and maintain normal NMDA receptor function and synaptic plasticity. This state may co-occur with cognitive impairment, particularly in the domain of immediate memory. However, the cross-sectional design precludes distinguishing whether reduced Klotho is a cause or a consequence of the illness process in chronic schizophrenia. Whether Klotho acts independently or interacts with other factors within these regulatory pathways remains unclear and warrants further molecular investigation. To test this hypothesis and clarify the underlying mechanisms, future studies that combine longitudinal design with pathway-specific measurements would be needed to distinguish causality from mere association.

This study has several limitations. Our cross-sectional design doesn’t allow us to infer causality or track how Klotho changes over the course of illness. That would require longitudinal or interventional data. The sample size is modest, so some analyses may have been underpowered. We also lacked measures of oxidative stress, inflammatory markers, or other relevant pathophysiological indicators, so we couldn’t probe mechanisms. On top of that, we didn’t assess depressive symptoms, functional status, motivation, or other clinical variables that could influence cognitive performance in schizophrenia patients, which leaves some confounding uncontrolled. Inconsistencies in Klotho levels between peripheral blood and cerebrospinal fluid remain unclear (57, 58), warranting further validation through additional cohort studies. Finally, all our participants were Han Chinese, which helps control for genetic confounding but means we don’t know whether the findings generalize to other ethnic groups.

## 5 Conclusion

In summary, patients with chronic SZ showed significantly reduced serum Klotho levels, with a significant association observed between these reductions and immediate memory. This finding suggests a potential contribution of the Klotho protein to cognitive dysfunction in SZ. Future large-scale, multicenter studies are needed to elucidate the underlying pathophysiological mechanisms and to determine whether Klotho may serve as a biomarker or therapeutic target for cognitive impairment in these patients.

## Data Availability

The raw data supporting the conclusions of this article will be made available by the authors, without undue reservation.
