# Supplementary material for: Reduced serum Klotho in chronic schizophrenia: diagnostic value and association with cognitive impairment
Source: Front Psychiatry. 2026 Aug 11;17:1918894. doi: 10.3389/fpsyt.2026.1918894 (PMC13503314; doi:10.3389/fpsyt.2026.1918894)
Supplement: Supplementary file 1 [file Supplementaryfile1.docx]

**Supplementary Figure 1.** Serum Klotho levels stratified by sex and smoking status in patients with schizophrenia and healthy controls.

Data are presented as mean ± standard deviation. Comparisons between males and females and between smokers and nonsmokers were performed using unpaired *t*-tests. No statistically significant differences were observed within either cohort (all *P* > 0.05). HCs: Healthy controls; SZ: Schizophrenia.

**Supplementary Table 1.** Baseline demographic and clinical characteristics of patients with schizophrenia in the monotherapy and polytherapy subgroups

| **Subgroup characteristics** | **Monotherapy group**  ***n* = 50** | **Polytherapy group**  ***n* = 58** | **Statistics** | ***P*** |
| --- | --- | --- | --- | --- |
| Gender (Male/Female) | 14/36 | 20/38 | *χ²* = 0523 | 0.469 |
| Age (years) | 42.12 ± 10.63 | 43.26 ± 8.84 | *t* = −0.608 | 0.545 |
| BMI (kg/m²) | 24.80 ± 4.24 | 24.82 ± 3.97 | *t* = −0.027 | 0.979 |
| Smoking status (Yes/No) | 5/45 | 12/46 | *χ²* = 2.313 | 0.128 |
| Disease duration (months) | 186.0 (120.0, 303.0) | 180.0 (108.0, 252.0) | *Z* = −0.630 | 0.529 |

Data are presented as mean ± standard deviation (SD), median (interquartile range, IQR), or number of participants (*n*), as appropriate. Categorical variables were compared using the chi-square test, normally distributed continuous variables using the independent samples *t*-test, and non-normally distributed continuous variables using the Mann–Whitney *U* test. BMI: Body mass index.

**Supplementary Table 2.** Comparison of serum Klotho levels among patients from four participating psychiatric hospitals

| Hospital | *n* | Klotho (pg/mL), Median (IQR) | Mean Rank |
| --- | --- | --- | --- |
| 1 | 39 | 242.64 (82.24) | 59.36 |
| 2 | 17 | 216.14 (71.19) | 50.82 |
| 3 | 28 | 216.53 (80.87) | 51.00 |
| 4 | 24 | 215.04 (70.99) | 53.29 |

Data are presented as median (interquartile range). Hospitals are coded as follows: 1 = The Fourth People’s Hospital of Lianyungang; 2 = Lianyungang Rehabilitation Hospital; 3 = Ganyu District Rehabilitation Hospital of Lianyungang; 4 = Guanyun County Psychiatric Hospital of Lianyungang. Comparisons across hospitals were performed using the Kruskal-Wallis one-way analysis of variance, which revealed no significant difference (*H* = 1.558, df = 3, *P* = 0.669).
